# Overcoming Drug Resistance in Advanced Prostate Cancer by Drug Repurposing

**DOI:** 10.3390/medsci10010015

**Published:** 2022-02-18

**Authors:** Hisham F. Bahmad, Timothy Demus, Maya M. Moubarak, Darine Daher, Juan Carlos Alvarez Moreno, Francesca Polit, Olga Lopez, Ali Merhe, Wassim Abou-Kheir, Alan M. Nieder, Robert Poppiti, Yumna Omarzai

**Affiliations:** 1 Arkadi M. Rywlin M.D. Department of Pathology and Laboratory Medicine, Mount Sinai Medical Center, Miami Beach, FL 33140, USA; juan.alvarez@msmc.com (J.C.A.M.); fmpolit@gmail.com (F.P.); robert.poppiti@msmc.com (R.P.); yumna.omarzai@msmc.com (Y.O.); 2 Division of Urology, Columbia University, Mount Sinai Medical Center, Miami Beach, FL 33140, USA; timdemus@gmail.com (T.D.); alan.nieder@msmc.com (A.M.N.); 3 Department of Anatomy, Cell Biology, and Physiological Sciences, Faculty of Medicine, American University of Beirut, Beirut 1107-2020, Lebanon; maya.moubarak@u-bordeaux.fr (M.M.M.); wa12@aub.edu.lb (W.A.-K.); 4 CNRS, IBGC, UMR5095, Universite de Bordeaux, F-33000 Bordeaux, France; 5 Faculty of Medicine, American University of Beirut, Beirut 1107-2020, Lebanon; dkd04@mail.aub.edu; 6 Herbert Wertheim College of Medicine, Florida International University, Miami, FL 33199, USA; olope070@med.fiu.edu; 7 Department of Urology, Jackson Memorial Hospital, University of Miami, Leonard M. Miller School of Medicine, Miami, FL 33136, USA; ali.merhe@jhsmiami.org

**Keywords:** drug repurposing, androgen-deprivation therapy, prostate cancer, CRPC

## Abstract

Prostate cancer (PCa) is the second most common cancer in men. Common treatments include active surveillance, surgery, or radiation. Androgen deprivation therapy and chemotherapy are usually reserved for advanced disease or biochemical recurrence, such as castration-resistant prostate cancer (CRPC), but they are not considered curative because PCa cells eventually develop drug resistance. The latter is achieved through various cellular mechanisms that ultimately circumvent the pharmaceutical’s mode of action. The need for novel therapeutic approaches is necessary under these circumstances. An alternative way to treat PCa is by repurposing of existing drugs that were initially intended for other conditions. By extrapolating the effects of previously approved drugs to the intracellular processes of PCa, treatment options will expand. In addition, drug repurposing is cost-effective and efficient because it utilizes drugs that have already demonstrated safety and efficacy. This review catalogues the drugs that can be repurposed for PCa in preclinical studies as well as clinical trials.

## 1. Introduction

### 1.1. Prostate Cancer

In 2022, the United States will have an estimated 268,490 new cases of prostate cancer (PCa) and 34,500 related deaths [1]. Nearly 95% of PCa cases are adenocarcinomas, and 70 to 85% are located in the periphery of the gland [2]. Such tumors tend to extend beyond the prostatic tissue and invade the local perineural space; this histologic finding by itself, though, does not predict positive surgical margins, extraprostatic extension, seminal vesicle invasion, or positive lymph nodes [3]. Lymphovascular invasion, however, was shown to be associated with positive surgical margins, extracapsular extension, seminal vesicle invasion, positive lymph nodes, biochemical recurrence, and decreased overall survival [4]. Metastasis occurs most commonly in regional lymph nodes and bone, followed by lung, then liver [5].

Standard PCa screening consists of measuring the serum prostate-specific antigen (PSA) level and performing a digital rectal exam (DRE); recently, multiparametric magnetic resonance imaging (MRI) has emerged as an additional screening tool [6]. Combining these data with background information, such as family history and race/ethnicity, a shared-decision is usually made between the patient and the urologist on whether or not to pursue prostate biopsy. Prostate biopsy is conducted transperineally or, more commonly, transrectally under ultrasound guidance (TRUS), and it typically consists of 12-core tissue samples that target medial- and lateral- base, mid, and apex regions on both the left and right lobes [7,8].

Clinical staging of PCa combines data from a DRE and needle biopsy results. Though clinical stage correlates closely with pathological stage, pathological stage is the most definitive predictor of biochemical recurrence [9]. Treatment primarily consists of active surveillance (AS), surgery, or radiation, with androgen deprivation therapy (ADT) and chemotherapy used as adjuvants in high-risk disease. AS is a conservative approach considered for patients with Grade group 1 (3 + 3) or 2 (3 + 4) with PSA < 10 ng/mL and involves close monitoring through quarterly PSA tests and an annual prostate biopsy [10]. Patients with Grade group 3 or higher are indicated for definitive therapies: surgery or radiation. High-risk patients, PSA > 20 ng/mL or Grade groups 4 or 5, who choose radiation should receive adjuvant ADT with GnRH agonists or antagonists [11]. High-risk patients with metastasis are managed with ADT and antiandrogens or ADT and docetaxel [12].

**Figure 1:**
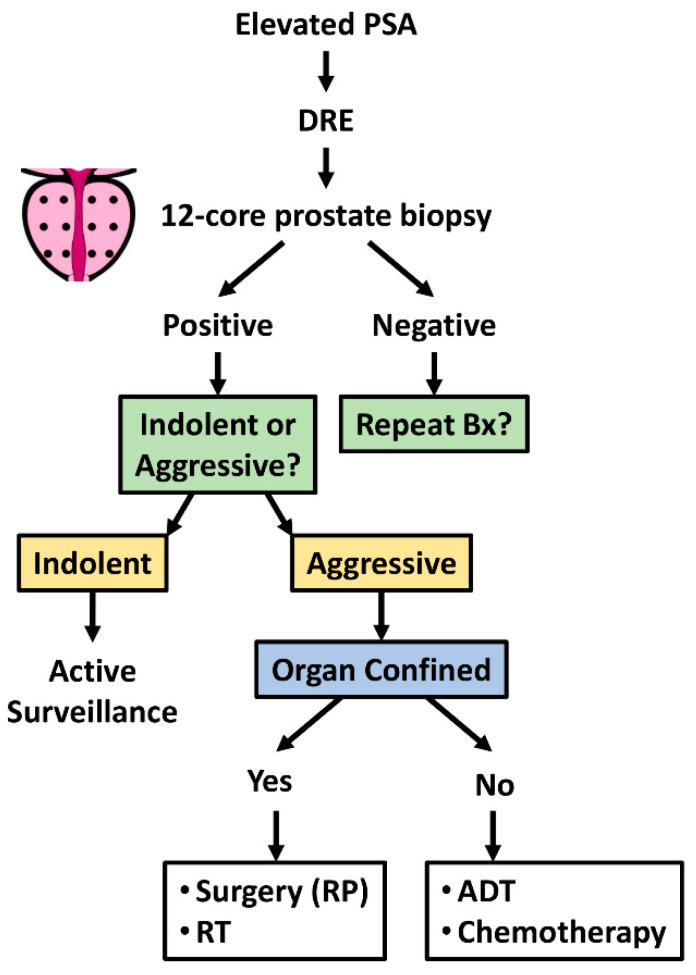
Clinical diagnosis, patient stratification, and treatment options for prostate cancer. Schematic of the clinical diagnosis and patient stratification for appropriate PCa treatment. Abbreviations: ADT: androgen deprivation therapy; Bx: biopsy; DRE: digital rectal examination; PSA: prostate specific antigen; RP: radical prostatectomy; RT: radiation therapy.

Following definitive treatment, 20 to 30% of patients develop biochemical recurrence (BCR) [13,14]. The latter marks the relapse of disease, defined as PSA > 0.2 ng/mL following RP or nadir PSA + 2.0 ng/mL after radiation [12]. Although tumor regression and remission follow ADT, resistance frequently occurs, and in many patients, it is inevitable [15,16]. Resistance to ADT is referred to as castration resistant prostate cancer (CRPC) or hormone-refractory PCa (HRPC). Clinically, patients can progress from non-metastatic CRPC to metastatic CRPC (mCRPC). The role of androgen signaling, as well as other signaling pathways, is important in understanding the mechanisms for CRPC, and using this knowledge facilitates the opportunity for drug targets (Figure 1).

### 1.2. Therapy Resistance in Prostate Cancer

Physiologically, testosterone passively diffuses into prostate cells where it either directly binds to the androgen receptor (AR) or is converted to dihydrotestosterone, which binds the AR with higher affinity. Bound AR undergoes a conformational change with a dissociation of heat shock proteins, followed by a nuclear translocation, where it dimerizes and serves as a transcription factor that regulates gene expression. In embryology, the prostate does not mature without androgens and becomes atrophic, as seen in individuals with 5 alpha reductase deficiency [17]. The low levels of exposure to androgens are, in fact, protective against development of PCa. The vital role androgens play in PCa was first described by Huggins who showed that prostate tumors regress by removing the primary source of testosterone through orchiectomy [18]. However, deprivation of serum testosterone initially leads to low DHT levels, which is the primary mediator of AR signaling. Withdrawal of these ligands leads to dramatic apoptosis of androgen-dependent tumor cells, leaving a subset of androgen-independent cells in a dormant state [19], some of which have cancer stem cell (CSC) properties. Additionally, ADT has been shown to alter the dynamics of the prostate tumor microenvironment, affecting stromal, endothelial, and immune cells, as well as promoting epithelial-to-mesenchymal transition, which is believed to be a contributor to therapy resistance in PCa [20,21].

There are various methods for PCa cells to achieve resistance, and some are specific to the modality of androgen deprivation. For example, leuprolide, a GnRH agonist that has been a first line treatment for the last 20 years in ADT, inhibits synthesis of androgens by removing the pituitary’s signal to make testosterone. The tumor cells escape by making their own testosterone in an autocrine fashion. By mutations that drive AR gene amplifications or in certain predisposing polymorphisms (such as short CAG sequences found more often in AA men), the AR becomes more sensitive and can respond to minimal intratumoral levels of testosterone [22]. Abiraterone, a relatively novel agent, can block intratumoral testosterone production, but PCa cells have various routes of resistance. For example, gain-in-function mutations in the AR allow it to use other molecules as ligands, such as those downstream of glucocorticoid or IL-6 transmembrane signaling, in order to translocate into the nucleus and cause gene expression [23]. Enzalutamide, one of the strongest anti-androgen agents, can block AR’s promiscuity with other molecules; however, PCa cells are resilient and eventually continue proliferating, even in the absence of AR activity.

### 1.3. Molecular Landscape in Prostate Cancer

The development of androgen-independent PCa is tightly connected to dysregulations within intracellular signaling cascades. Commonly studied pathways in PCa include PI3K/AKT/mTOR, Ras/Raf/MEK/ERK (MAPK), and WNT/β-catenin. For example, a *PTEN* gene deletion, found in 16 to 32% of PCa, results in increased mTOR signaling, and increased mTOR signaling is correlated with poor survival in PCa [24]. Likewise, activation of MAPK/ERK signaling, through various mechanisms, has demonstrated resistance to Enzalutamide [25]. WNT signaling dysregulation, either in a β-catenin dependent- or independent-fashion, can also drive aggressive PCa [26]. 

More important is the cross-communication between different cell signaling pathways in PCa growth and progression. For example, prostate cells with isolated mutations in the *Ras/Raf/MEK/ERK* signaling are unable to initiate tumorigenesis. However, when combined with alterations to *PI3K/AKT/mTOR* signaling, such as a *PTEN* deletion, *RAS* mutations induce tumorigenesis and facilitate epithelial-to-mesenchymal transition (EMT), making prostate tumors substantially more aggressive [21,27]. Mutations in the Ras pathway can also facilitate resistance to medications targeted towards PI3K/AKT/mTOR pathway [28]. Therefore, the co-administration of drugs targeting multiple signaling pathways can be beneficial. This was demonstrated in a mouse model, where the co-administration of Rapamycin (an inhibitor of mTOR) and Mirdametinib (an inhibitor of MEK) inhibited hormone-refractory PCa cell growth, demonstrating the interdependence of the Ras and AKT signaling cascades in advanced PCa [29].

Another example of dependent cross communication is seen with the gene fusion *TMPRSS2-ERG* (present in 40 to 80% of PCa) [30,31]. As with isolated *Ras/Raf/MEK/ERK* mutations, *ERG* oncogene requires signaling aberrations from other pathways—such as *FOXO1* deletions from *AKT* dysregulation—to have significant tumorigenic and invasive features [32].

Cross communication between aberrant cell signaling pathways is also paramount to the emergence and maintenance of PCa stem cells (PCSCs) in an androgen-resistant tumor state [33]. Studies have shown that interactions between hypoxia induced factors (HIFs) and *PI3K*, *MAPK*, or *WNT* maintain PCa cell “stemness” [34]. In fact, ADT directly drives the release of hypoxia inducible factors [35]. The presence of PCa stem cells might explain the heterogeneous cell populations seen in prostate tumors. PCSCs are attractive drug targets, as more evidence points to these cells as being the origin of CRPC [33]. Therefore, studies such as our own are necessary to explore available United States Food and Drug Administration (FDA)-approved drugs that can inhibit or slow the progression of the disease.

Drug repurposing has always been an opportunistic and fortuitous procedure [36,37]. For example, sildenafil citrate, often known as “Viagra”, was first discovered as an antihypertensive medication, then repurposed by Pfizer for the treatment of erectile dysfunction, based on retrospective clinical experience [38,39]. Furthermore, thalidomide, which was formerly used to treat morning sickness in pregnant women, was pulled from the market because of its known association with severe bone birth abnormalities in children [40]. However, serendipity led to the repurposing of thalidomide for the treatment of erythema nodosum leprosum (ENL) [41] and later, in multiple myeloma [42]. Currently, the global SARS-CoV-2 (COVID-19) outbreak has given the medication repositioning strategy a new sense of urgency [43,44,45,46]. Traditional drug research is lengthy, owing to the regulations of creating new medications and treatments. Therefore the quicker repositioning technique has piqued attention to identifying compounds that could counteract the consequences of the viral infection including chloroquine [47], hydroxychloroquine [48], enalapril [49], and remdesivir [47], in addition to others [45].

## 2. Repurposing Approved Drugs in Cancer

### 2.1. Introduction to Drug Repurposing

Drug repurposing, also known as drug repositioning, reprofiling, or retasking, is a strategy for finding new uses for approved or investigational pharmaceuticals beyond their original medical indication [41]. Compared to de novo drug development, drug repurposing offers various advantages over developing a new drug for a specific application. Usually, speeding up pharmaceutical research and development is frequently linked to an increase in the development risk. Repositioning candidates have typically undergone research in preclinical models and human clinical trials, as well as safety assessment, optimization, and sometimes, formulation development and, hence, have well-known safety and pharmacokinetic properties. As a result, the time frame for drug development can be greatly reduced, along with the rate of failure in later efficacy trials [38,41]. Relative to the stage of development of the repurposing candidate drug, drug repurposing requires less investment in terms of preclinical and phase I and II expenditures, but the regulatory and phase III costs may be comparable to those for a new drug in the same indication [38,50]. Together, these advantages add up to lower risk and faster return on investment in the development of repurposed pharmaceuticals, as well as lower average associated costs if failures are included. 

### 2.2. A Computational Approach for Drug Repurposing

Computational methods, also known as in silico drug repurposing [51], are critical in predicting novel indications for current medicines, and they largely rely on data from databases such as DrugBank [52], ChemBank [53], Genecards [54], OMIM [55], and PubMed [56]. As a result of the collection and systematic analysis of diverse data, including gene expression, chemical structure, genotype or proteomic data, or electronic health records (EHRs), innovative approaches for drug repositioning can be developed by emphasizing possible candidates for drug-disease connections [57]. Here, we will discuss the most prevalent computational techniques as well as some examples of medication repurposing.

#### 2.2.1. Network-Based Drug Repurposing

Following the advancements in genotyping technology, reduced genotyping costs, and the completion of the Human Genome Project, genome-wide association studies (GWAS) have been performed to find genetic variants that affect common diseases [58]. Using GWAS, new targets that might be shared across the medicines and disease phenotypes examined could be identified, leading to therapeutic repurposing [59,60,61]. In some instances, the genes identified in a GWAS research are not druggable targets. However, a network-based method may reveal genes that are upstream or downstream of the GWAS-associated target and might be used for repurposing [38,62]. The network-based method that integrates information on medicines and their druggable targets constructs drug-disease networks based on various data, or indirectly inferred using computational algorithms, to predict new drug candidates [58,63].

Network-based clustering algorithms were employed to discover subnetworks that allow for prospective candidate drug–target connections [64,65,66]. For instance, vismodegib, a known inhibitor of the Hedgehog signaling pathway, was identified to treat Gorlin syndrome [67], and iloperidone, an antipsychotic for the treatment of schizophrenia, was identified as a novel drug for hypertension [65]. Similarly, network-based propagation techniques are utilized to discover prospective candidate medicines. A random walk propagation algorithm expands a set of disease-associated genes to genes sharing neighbors in a protein-protein network or gene-gene network. Using this strategy, several medicines were identified for novel indications, such as donepezil for Parkinson’s disease, methotrexate for Crohn’s disease, gabapentin for anxiety disorder, risperidone for obsessive-compulsive disorder, in addition to cisplatin for breast cancer [68]. 

Functional genomics is also widely used to map cancer dependencies and identify therapeutic targets in cancer. This includes genetic screens based on CRISPR/Cas9 technology [69,70,71], reverse genetic screens, and chemogenomic screens [72]. In PCa, this technology has proven to be crucial for clinicians in their decision-making regarding patient treatment, especially in the era of precision medicine. For instance, suppression of cyclin-dependent kinase 12 (CDK12)—which is essential for PCa cell survival—by the covalent inhibitor THZ531 had an anti-PCa effect by downregulating androgen receptor signaling [70]. Another study using chemogenomic screening demonstrated that Hsp70 co-chaperone DNAJA1 is a hub for anticancer drug resistance [72].

#### 2.2.2. Profile-Based Drug Repurposing

Profile-based drug repurposing serves for the comparison of a drug with another drug, disease, or clinical phenotypes using different profiles of the drug to be repurposed [38,73,74]. An example is the expression-based profile, also known as a transcriptome (RNA) signature, which allows for drug-drug similarity and drug-disease similarity [75,76]. The transcriptome signature of a repurposed medication is established by comparing differential gene expression in a cell or tissue before and after therapy, which is then compared to the disease-associated expression profile. For instance, if the drug can reverse the expression pattern of a given set of genes in a particular disease to be closer to that obtained for the healthy state, then that drug might be able to revert the disease phenotype itself. As a result, this computational technique employs the signature reversion principle (SRP), which allows for the comparison of transcriptomic profiles between drugs and diseases and helps in anticipating whether the repurposed drug may be effective on the disease of interest [38,77,78]. Additionally, this concept uncovered novel repurposing prospects in a variety of therapeutic domains [79,80,81,82] in addition to chemo-sensitizers in lymphoid malignancies [83]. Furthermore, the expression-based profile concept requires publicly available gene expression databases. The Broad Institute’s Connectivity Map (cMap) is derived from the outcomes of treating numerous cell types with over 1300 compounds [84]. However, to make cMap more effective, this resource may be coupled with other public sources of gene expression data, including Gene Expression Omnibus and Array Express [38].

The second type of profile-based approach is the structure-based computational strategy. Since drugs of similar chemical structures may share common biological activity, this strategy relies on the drug chemical structures to compare between drugs and identify the new drug-target association [85,86]. Using a similarity ensemble approach (SEA), Keiser and colleagues identified 23 new drug–target associations [73]. Another technique used is molecular docking to identify the ligand-receptor association [87]. Conventional docking, for example, involves evaluating numerous ligands (drugs) against a known receptor in a particular disease. Reverse docking, on the other hand, tests drug libraries against a wide spectrum of receptors to find potential interactions [38,88]. Using a high-throughput computational docking technique, Dakshanamurthy and colleagues have reported that mebendazole, an antiparasitic drug, has the structural capacity to block vascular endothelial growth factor receptor 2 (VEGFR2) [89].

However, this technique is limited due to the lack of 3D structures for some protein targets, lack of macromolecular target databases, a high rate of false positives, some predictability limitations, as well as the questioned docking algorithms utilized due to variances in software packages [38,76]. Nonetheless, recently, a new promising tool, “AlphaFold”, was developed, which counters this issue of structure prediction. It is a neural network-based computational model that can predict protein structures with atomic accuracy [90]. AlphaFold can be used for molecular replacement and for interpreting cryogenic electron microscopy maps. Importantly, developing accurate protein structure prediction algorithms will accelerate the advancement of structural bioinformatics to keep pace with the genomics revolution.

Furthermore, profiling based on side-effect similarities is another resource for computational drug repurposing. Similar side effects between two different drugs are considered to indicate shared physiology, suggesting that they may have a similar mode of action by affecting the same target or pathway [91]. It is also possible that a drug’s impact is comparable to that of a certain illness, indicating common drug-disease physiology [92]. Although this is a good technique for identifying repurposing possibilities, the lack of well-defined adverse effect profiles may restrict its application [76]. On the other hand, artificial intelligence technologies capable of text mining and natural language processing may offer future opportunities to overcome these constraints [93]. 

#### 2.2.3. Data-Based Drug Repurposing

Some drug repurposing discoveries were made through simple clinical studies or pharmacological analyses, rather than a systematic review of clinical data. Examples of these discoveries are sildenafil for erectile dysfunction [41], aspirin for colorectal cancer [94], raloxifene in breast cancer, and propranolol in osteoporosis [95].

Retrospective clinical data is becoming increasingly popular to discover drug repurposing possibilities [96]. Electronic health records (EHRs) are not only a rich source of laboratory test results and prescription information for patients, but they are also a source of indications for drug repurposing [96,97]. Furthermore, the massive volume of EHR data provides large statistical power [57]. Paik and colleagues have employed this approach to extract clinical data, including laboratory tests and genetic fingerprints, to find over 17,000 known drug-disease correlations and identified terbutaline sulfate as a potential option for the treatment of amyotrophic lateral sclerosis (ALS) [97]. However, obtaining and using EHR data remains challenging due to ethical and legislative hurdles that may limit data access, as well as the difficulty of extracting the unstructured data included in these databases [96].

Text-mining tools, another form of data-based medication repurposing strategy, allows for prioritizing prospective research areas and expedite the disease-drug repurposing process among the extensively accumulating scientific literature [98]. Thus, to discover possible drug-disease connections in the literature, several text mining approaches have been used. In this regard, Li and colleagues have developed disease-specific drug-protein connectivity maps for Alzheimer’s disease through combining gene/protein and drug connection information based on protein interaction networks and literature mining. Their approach revealed diltiazem and quinidine as possible therapeutic candidates for Alzheimer’s disease [99]. A method to construct sentence graph networks was achieved using text mining techniques to find new sarcoidosis disease targets [100]. In addition, Kuusisto and colleagues introduced KinderMiner as a text mining approach for identifying possible indications of old medicines [98], while others published an algorithm to identify potential anti-Alzheimer’s disease drugs, target, and prioritize them, via systematic ‘omics’ mining [101]. 

### 2.3. Drug Repurposing Approach in Cancer

Lately, researchers and clinicians are exploring drug repurposing to overcome the shortage of medication for novel cancer treatments [102,103,104]. As such, repurposing drugs with tolerable side effects and known pharmacokinetics and pharmacodynamics profiles offers an alternative to standard anti-cancer chemotherapeutics [51]. Excellent prospects for drug repurposing have been offered for medicines that may have the ability to address more than one target, as detailed in the next section, thanks to advancements in genomic and proteomic tools. As a result, drug repurposing using non-oncology medications has been applied with medicines that are effective for cancer hallmarks but were not designed originally for cancer treatment.

Drug repurposing has many benefits compared to de novo drug studies. These advantages mainly revolve around time and cost, where a study by Kaitin et al. showed that it takes approximately 8.3 years for an anti-cancer medication to get approved, compared with 3 to 4 years for a repurposed drug [105]. Additionally, among the various advantages of drug repurposing is being cost-effective, where a repurposed drug is estimated to cost USD 300 million to reach market, compared with USD 2–3 billion for studying a new chemical entity [104,106].

## 3. Drug Repurposing in Prostate Cancer

### 3.1. Clinical Challenges in Prostate Cancer

A major issue with low-risk PCa is that it is frequently overtreated, whereas high-risk PCa is frequently undertreated. Many individuals with high-risk illnesses are only offered palliative androgen deprivation therapy (ADT) rather than intense local therapy. According to the CaPSURE database, ADT is given to 41% of high-risk patients, whereas RP and RT are given to 24% and 28% of high-risk patients, respectively [107,108]. In contrast, local therapy, with either RP or RT, depending on the classification of high-risk illness, results in a 49–80% progression-free probability (PFP) [109,110]. Furthermore, as compared to observation or ADT alone, numerous randomized studies have indicated that active treatment improves survival in individuals with high-risk PCa [111,112].

Another challenge is choosing the appropriate treatment along with the proper timing of administration. When androgen blocking is used as first-line therapy, for example, high response rates are attained; nonetheless, most men proceed to CRPC. Furthermore, systemic chemotherapies have been proven to improve clinical outcomes in individuals with hormone-refractory PCa. However, they are not curative [113].

Furthermore, despite early detection and intervention, advanced PCa might metastasize to lymph nodes and bones, lowering the quality of life and decreasing the median survival rate [114]. As a result, detecting bone metastases is crucial in clinical practice, since the beginning of bone metastasis frequently necessitates the start of chemotherapy and/or bone-targeted treatment [115,116,117]. Another point of contention in PCa care is the timing of hormonal therapy for patients with increasing PSA who have failed initial treatment, as well as if hormones can give an extra advantage to external beam radiotherapy [118].

On the other hand, some challenges in PCa are owed to the limited screening tools and their diagnostic accuracy. Prostate-specific antigen (PSA) is a widely accepted screening tool for PCa. However, elevated PSA levels may result when the normal architecture of the prostate is disrupted, such as in benign prostatic hyperplasia (BPH) and prostatitis. Therefore, PSA is a prostate-specific marker but not PCa-specific [119,120]. Although blood PSA levels associates with clinical stage and tumor phenotype, they are of little use in predicting stage for patients [113,119]. Other tests based on PSA derivatives are required to improve on standard blood PSA, such as PSA density, velocity, and age-specific reference range, and PSA isoforms such as free PSA, pro-PSA, and BPSA (benign PSA) [121,122]. Furthermore, while widespread PSA screening has resulted in early detection and a considerable reduction in PCa staging at diagnosis, it has also resulted in extra, likely unnecessary biopsies, as well as higher over-detection rates [113,121,123].

Moreover, the Gleason score is used to grade biopsies for PCa by histopathological examination of tumor development. Biopsies, on the other hand, may not match the prostatectomy specimen due to sampling issues, as well as the possibility of interobserver variability. In addition, people with morphologically similar PCa may behave differently due to inter- and intra-tumor heterogeneity [124].

Several techniques are available to help determine a patient’s life expectancy; however, their estimation accuracy is limited. Actuarial life tables, which are readily available, offer a fair estimate, but they do not take into consideration specific medical comorbidities [118]. Comorbidity indices, such as the Charlson comorbidity index, predict life expectancy depending on medical conditions; however, it may ignore or exaggerate the relevance of certain morbidities [125]. Nomograms, on the other hand, may offer more precise predictions with a prediction accuracy of 69–84% for life expectancy after therapy for localized PCa [126,127,128].

Also, false-positive and false-negative biopsies reduce the diagnostic accuracy of PCa [129]. Because of the inherent heterogeneity of PCa [130], current systematic biopsy methods done with TRUS guidance often result in underdiagnosis of PCa [131]. To address these limitations, more sensitive and selective serum–tissue biomarkers should be developed, in addition to conjugating new imaging technologies with standard TRUS, to increase biopsy sensitivity [113,132].

### 3.2. Drugs Repurposed in Prostate Cancer: From Benchside to Bedside

Conventional anti-cancer chemotherapeutic agents have well-known side effects that severely impair cancer patients’ quality of life. Therefore, drug repurposing is an effective alternative method for identifying new anti-cancer candidates from the existing pharmacological pool [51,133]. Here, we will discuss three main categories of drug repurposing studies for PCa based on different discovery and validation methods (Table 1).

**Figure 2:**
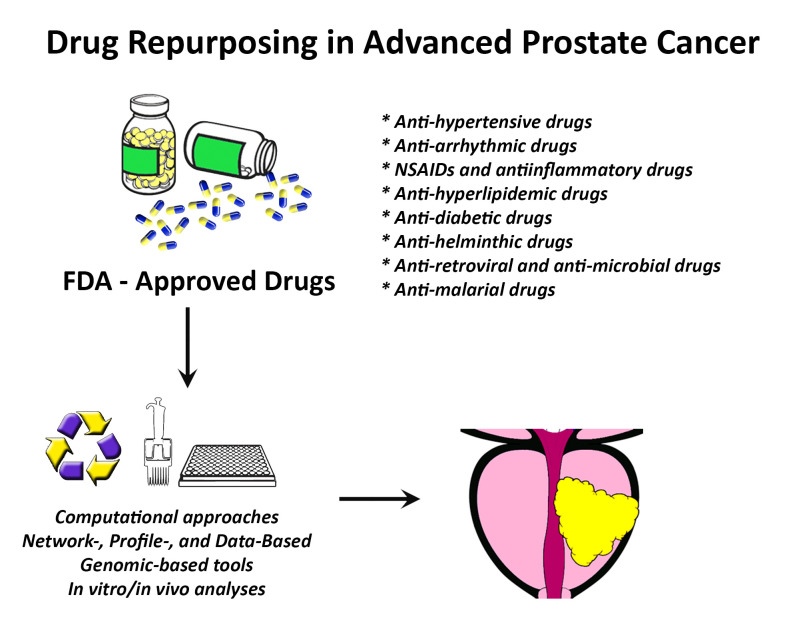
Drug repurposing in advanced prostate cancer. Using computational approaches and pre-clinical analyses, many Food and Drug Administration (FDA)-approved drugs could be identified and repurposed to treat patients with advanced prostate cancer. Abbreviations: FDA: U.S. Food and Drug Administration; NSAIDs: non-steroidal anti-inflammatory drugs.

The first category is classified based on the knowledge and ability of medical practitioners or researchers to organize scientific observations of random events. For instance, ormeloxifene, a clinically approved selective estrogen receptor modulator, exhibits anti-cancer properties in multiple cancers including ovarian, head and neck, and breast cancers. However, Hafeez et al. reported ormeloxifene–mediated inhibition of oncogenic β-catenin signaling and EMT progression in PCa, mainly by repressing N-cadherin, MMPs (MMP2 and MMP3), β-catenin/TCF-4 transcriptional activity, and inducing pGSK3β expression. In addition, treatment with ormeloxifene inhibited tumorigenic, metastatic, and invasive capacity of PCa cells in vitro and reduced prostate tumors in xenograft mouse models [134]. Naftopidil, a selective adrenoreceptor A1D antagonist, is used for treating lower urinary tract symptoms triggered by benign prostatic hyperplasia [135]. It is also shown to improve the efficiency of radiotherapy (RT) treatment in PC-3 xenograft models as compared with monotherapy with naftopidil or RT [136] (Figure 2).

**Table 1:** Summary table of the drugs that have been repurposed to be used in prostate cancer in pre-clinical models.

Ref.	Drug	Original Indication	PCa Cell Lines Targeted	In Vivo Studies	Mode(s) of Action	Effect(s)
[137]	Propranolol	Anti-hypertensive	-	-	Blockade of beta-2 receptors Inhibition of PAP of lipins	Halted PCa proliferation Promoted autophagy vesicles and hence cancer cell death
[138]	Digoxin	Anti-arrhythmic			Inhibition of Na^+^/K^+^ ATPase pump which increases intracellular Ca^2+^ levels	
[138,139]	Ouabain	Anti-arrhythmic	PPC-1	Male SCID		Sensitize PPC-1 to anoikis, normal apoptosis via detachment from ECM
[140]	Aspirin	Anti-inflammatory	LNCaP		Inhibition of cyclooxygenase (COX) pathway Decreased PG production Reduction of cyclin D1	Promote antitumerogenic effects in combination with statins
[141]	Celecoxib	Anti-inflammatory	LNCaP & androgen-nonresponsive PC-3	LNCaP in bovine brain extracts	Selective COX-2 blockade Blockade of Akt pathway	Induction of apoptosis
[142]	dexamethasone	Anti-inflammatory	DU145 PCa cells		Decreased production of inflammatory mediators Inhibit ERG, an oncogene	Decreased proliferation of PCa in an in silico model via ERG inhibition
[143,144,145]	Simvastatin	Anti-hyperlipidimic	PC3, 22Rv1, DU145, DU145R80, LNCaP prostate cancer cell lines and EPN normal prostate epithelial cells	DU145R80, 22Rv1 parental and docetaxel resistant cells in xenografts	Inhibition of HMG-CoA reductase	Sensitization of PCa cells to docletaxil in combination with valproic acid via YES inhibition Promotion of apoptosis via cholesterol depletion Sensitization of anti-androgen resistant CRPC
[146,147,148]	Metformin	Anti-diabetic			Increased sensitivity to insulin inhibiting hepatic production of glucose Restriction of hepatic gluconeogenesis	Decreased growth, proliferation and differentiation by downregulating PI3K axis Decreased ARs, acting as an adjunct to ADT
[149]	Glipizide	Anti-diabetic	PC-3, 22Rv1 and DU145 PC	TRAMP transgenic mouse model	Stimulation of β-cell insulin secretion	Inhibition of angiogenesis by targeting HMGIY/ANGPT1 pathway
[150]	Mebendazole	Anti-helminthic	LNCaP		Inhibition of microtubule assembly	Promoting cell death in synergism with docletaxel
[151]	Niclosamide	Anti-helminthic	LNCaP, VCaP, CWR22Rv1, PC3 and HEK293	CWR22Rv1 cells in SCID mice	Unclear; uncoupling of oxidative phosphorylation chain	sensitize treatment to enzalutamide by decreasing AR-V7
[152,153]	Nelfinavir	Anti-retroviral	DU145 and PC3 cell lines		Inhibition of proteases	Combatted CRPC by inhibiting Site-2 Protease (S2P) cleavage and Regulated Intramembrane Processing (RIP)
[154]	CMT-3	Anti-microbial	Many lines	xenografts of PC-3 tumors	Inhibition of protein synthesis	Promoted apoptosis in PCa cell lines via activation of caspase-3 and caspase-9
[155]	Zoledronic acid	Bisphosphonate	LuCaP 23.1, a PSA-producing human CaP xenograft	LuCaP 23.1, a PSA-producing human CaP xenograft	Inhibition of bone resorption via inhibition of osteoclast activity	Decreased metastatic potential in vivo and induced G1 arrest, proliferation decline and apoptosis in vitro
[156]	Valproic acid	Anti-epileptic	AR-positive (LNCaP and C4-2) and AR-negative (DU145 and PC3)	LNCaP, C4-2, and DU145 Xenograft models	Inhibition class I histone deacetylases (HDAC)	Reduced PCa cellular proliferation and upregulated caspase-2 and caspase-3 activity
[157]	Mifepristone	Anti-progestational steroid			Anti-progesterone with potent progesterone receptor (PR) affinity	Promotion of anti-cancer effects in both androgen sensitive and insensitive PCa, via induction of TFG-beta 1, a pro-apoptotic TF

Abbreviations: AR: androgen receptor; COX: cyclooxygenase; CRPC: castration-resistant prostate cancer; ECM: extracellular matrix; PCa: prostate cancer; PR: progesterone receptor; SCID: severe combined immunodeficiency.

Nitroxoline, a widely used antibiotic for treating urinary tract infections, inhibits endothelial cell proliferation for different solid cancer types [158,159]. It also mediates AMPK-dependent inhibition of the mTOR signaling pathway and cyclin D1-Rb-Cdc25A axis suggesting its potential role for therapeutic development against PCa [160]. Nelfinavir, a human immunodeficiency virus (HIV) protease inhibitor authorized by the FDA, is typically used in therapies for HIV patients [161]. Studies have identified certain nitroxoline-mediated anti-cancer mechanisms, such as inhibition of (PI3K)/Akt signaling pathway, the proteasome, and HIF-1α which limit angiogenesis, as well as activation of endoplasmic reticulum (ER) stress, autophagy, and apoptosis [162]. Interestingly, nelfinavir shows promising therapeutic potential for the treatment of CRPC through blocking site-2 protease cleavage [153].

Second are the drugs that have been evaluated in assays and categorized according to their activity. Itraconazole, the antifungal drug, which inhibits angiogenesis and the Hedgehog signaling pathway, has been tested in phase II clinical trials and found effective in men with metastatic CRPC. In addition, digoxin was repurposed for PCa treatment as it inhibits HIF-1α synthesis and tumor growth and was clinically trialed in patients with recurrent PCa [163,164]. Clofoctol, an antibiotic used to treat upper respiratory tract infections, is a promising inhibitor of PCa. Wang and colleagues reported that clofoctol reduced the development of PCa cells at clinically feasible doses. It also induced ER stress and activated all three Unfolded Protein Response (UPR) pathways, resulting in indirect suppression of protein translation and subsequent reduction in the amounts of G1/S cyclins, resulting in G1 cell cycle arrest. Furthermore, clofoctol was found to be effective in a PCa xenograft model in animals, making it a promising anti-PCa medication candidate suitable for human testing [165]. Several fatty acid synthase (FASN) inhibitors, including antifungal agent cerulenin, its synthetic derivative C75, and triclosan have been shown to inhibit cancer cell growth by inducing cell death. However, their evaluation in clinical trials was challenged due to pharmacological limitations [166,167]. Triclosan (TSC) is an approved bactericide in personal hygiene products that possesses a good safety profile, oral bioavailability, and stability in plasma [168]. Triclosan was found to be a superior alternative to C75 and orlistat in triggering cell death in PCa cells via the inhibition of FASN. In addition, it induced G0/G1 cell cycle arrest and dose-dependent reduction in total lipid content of PCa cells [167]. As a result, TCS-mediated suppression of the metabolic oncogene FASN has the potential to be developed as a treatment for advanced PCa.

Other drugs are classified using the in-silico drug repurposing approach. For example, risperidone, an antipsychotic agent, is used for the treatment of central nervous system disorders owing to its binding affinity for dopamine D2 and serotonin 5-HT2 receptors [169]. On the other hand, 17-β-hydroxysteroid dehydrogenase 10 (17HSD10) is responsible for Alzheimer’s disease pathogenesis and PCa cell survival upon ADT. Since risperidone targets 17HSD10, it is a possible treatment choice for both Alzheimer’s disease and PCa [170].

Furthermore, Zenarestat, an aldose reductase inhibitor, may be an effective cancer chemotherapeutic drug since aldose reductases promote tumor development by activating the transcription of NF-kB and AP-1 [171]. Based on thorough gene expression profiling and disease-gene-drug association data, zenarestat was repurposed to serve as a potential medication for treatment in PCa [172].

#### 3.2.1. Anti-Hypertensives and Anti-Arrhythmic Drugs

Beta-blockers act by blockage of beta-adrenergic receptors in the body and have been traditionally utilized for their anti-hypertensive and anti-arrhythmic properties [173]. One such beta blocker is the non-selective propranolol, which has been shown to exhibit anticancer properties, encompassing PCa [137]. Specifically, it has been shown to halt PCa proliferation and induce apoptosis, in synergism with a glucose analog, 2-deoxy-d-glucose (2DG). Propranolol also acts via inhibition of phosphatidic acid phosphatase (PAP), which has been shown to be overexpressed in certain cancers [174]. After induction by 2DG, propranolol’s blockade of PAPs leads to accumulation of LC3-II and p62, markers of autophagy, thus promoting cancer cell death [139].

Cardiac glycosides, such as digoxin and ouabain, exert their anti-arrhythmic properties via Na^+^/K^+^ ATPase pump inhibition, which increases intracellular Ca^2+^ levels [139]. Simspon et al. showed that ouabain sensitized resistant prostate adenocarcinoma cells (PPC-1) to aniokis, the process by which normal cells undergo apoptosis by detaching from the extracellular matrix (ECM) via a caspase-dependent mechanism [138].

Prazosin is a selective inhibitor of the alpha-1 adrenergic receptor and has been authorized for the treatment of hypertension [175]. Additionally, it is used to treat a variety of clinical conditions, including benign prostatic hyperplasia, Raynaud’s illness, and congestive heart failure [175,176,177]. In patient-derived glioblastoma-initiating cells (GICs), prazosin mediated growth inhibition through inhibiting the PKCδ- dependent AKT signaling pathway compared to neural stem cells that lack PKCδ [178]. Interestingly, prazosin has been shown to display antiproliferative activity, superior to that of other α1-blockers, by inducing G2 checkpoint arrest and subsequent apoptosis in PCa cell lines [179].

#### 3.2.2. NSAIDS, Anti-Inflammatory Drugs and Aspirin

Non-steroidal anti-inflammatory drugs (NSAIDs) act via inhibition of the cyclooxygenase (COX) pathway, thus limiting prostaglandin (PG) synthesis, and are one of the most widely used drugs for their anti-inflammatory, antipyretic, and analgesic effects [180]. NSAIDs have been shown to affect the proliferative, apoptotic, resistance, and metastatic potential of many PCa cell lines via both COX-dependent and independent mechanisms. The aforementioned results highlight its potential for use in therapy-resistant PCa [181]. An example of the COX-independent mechanism is demonstrated in the antitumorigenic effects of statin and aspirin combination on LNCaP cells, which are androgen-sensitive human PCa cells, via reduction in cyclin D1 levels [140]. Celecoxib, a selective COX-2 inhibitor, blocks Akt phosphorylation and activation, which, in turn, leads to apoptosis in PCa cells [141]. Another anti-inflammatory agent, the glucocorticoid dexamethasone, inhibited ERG activity, an oncogenic transcription factor, of prostate tumor cells in an in-silico model [142].

According to clinical studies, patients undergoing long-term NSAID treatment have a decreased chance of acquiring cancer [182,183]. Indomethacin, an NSAID used for rheumatic disease treatment [184,185], is a notable antineoplastic agent. Mechanistically, it inhibits Cox-1/2-dependent angiogenesis [186,187] as well as MAPK pathways [188]. It also inhibits cancer cell growth via impairing PKC-p38-DRP1 axis-dependent mitochondrial dynamics or downregulating Wnt/β-catenin signaling [189]. A clinical trial is currently ongoing (ClinicalTrials.gov; NCT02935205) to study the combined effect of hormone therapy using enzalutamide and indomethacin on PCa by lowering the amount of androgen the body makes and/or blocking the use of androgen by the tumor cells.

#### 3.2.3. Anti-Hyperlipidemic Drugs

Statins act as lipid-lowering agents by inhibiting the HMG-CoA reductase enzyme and thus inhibiting the rate-limiting step cholesterol biosynthesis [190]. However, preclinical and clinical evidence show that statins exhibit anti-neoplastic activity in various cancers [191,192]. Ianelli et al. demonstrated the synergistic effects of simvastatin and valproic acid in sensitizing PCa to docletaxel and decreasing resistance rates. This combination method targets the CSCs by inhibiting the action of the Yes-associated protein (YAP) oncogene, a transcriptional regulator implicated in many cancers, including PCa [143,144]. Zhuang et al. demonstrated that simvastatin acts to promote apoptosis via cholesterol depletion. Specifically, simvastatin acts via depletion of cholesterol-containing lipid rafts of PCa cells, which, in turn, inhibits protein kinase B (also known as AKT) signaling pathway [193]. Statins also have a role in sensitizing PCa cells to ADT. Kong et al. have shown that simvastatin treatment helps to re-sensitize PCa cell lines that have developed resistance to enzalutamide, an FDA approved anti-androgenic agent for the treatment of CRPC [145].

In a retrospective cohort study of men who underwent prostate biopsy, statin decreased risk of PCa and resulted in a better prognosis and reduction in PCa volume and PSA levels [194]. Other studies have indicated that statins reduce PCa-related mortality and risk [195,196], while others have found no impact [194]. Inconsistent clinical results make a definite link between statin usage and PCa difficult to establish [197].

#### 3.2.4. Anti-Diabetic Drugs

Metformin, a member of the biguanide family, acts as an anti-diabetic agent by increasing sensitivity to insulin, inhibiting hepatic production of glucose, and restricting hepatic gluconeogenesis [198]. Aside from its anti-diabetic properties, metformin has shown the potential to be used for its anti-cancer properties, particularly in PCa. Metformin inhibits the mitochondrial complex I of the electron transport chain, which leads to an increase in adenosine monophosphate-activated protein kinase (AMPK), in turn leading to the inhibition of the mammalian target of rapamycin (mTOR) and activation of tuberous sclerosis complex-2, a tumor suppressor gene [146]. By decreasing the levels of insulin in circulation, metformin downregulates phosphoinositide-3-kinase (PI3K) axis, a main contributor to cell growth, proliferation, and differentiation [147]. Furthermore, metformin has been shown to decrease androgen receptor expression, which could establish its role as an adjunct to ADT [148]. Glipizide, another antidiabetic drug that stimulates beta-cell insulin secretion, has shown tumor suppressive effects of PCa cells in a TRAMP transgenic mouse model. It does so mainly via inhibition of angiogenesis, particularly by targeting HMGIY/ANGPT1 signaling pathway [149].

Metformin also has significant anti-cancer activity [199,200,201] in breast, prostate, lung, cervix, ovarian, and CNS cancers, and it is being used in phase 1–4 clinical trials for cancer therapy [202]. Metformin reduced the risk of PCa diagnosis in comparison to other oral hypoglycemics [203]. In xenograft models, the combination of metformin and chemotherapy inhibits tumor development and prevents recurrence of PCa cells via inhibiting inflammatory pathways [204]. In addition, metformin and valproic acid (VPA) synergistically suppressed proliferation in both LNCaP (androgen dependent) and PC-3 (androgen independent) cell lines and enhanced apoptosis in patient-derived prostate tumor explants [205].

Long-term metformin therapy has also been found to significantly reduce the incidence of breast cancer in women with type 2 diabetes [206]. By reducing elevated insulin levels, metformin inhibits the growth of tumors expressing insulin receptors [207,208]. Metformin has also been shown to activate AMPK, a critical energy sensor in cellular metabolism, and to limit cancer cell proliferation via the negative regulation of mTOR needed for tumor survival [209].

#### 3.2.5. Anti-Helminthic Drugs

Mebendazole is a microtubule inhibiting drug that fights parasitic infections. Docetaxel is a treatment option for metastatic PCa; however, it had a modest effect on increasing the median survival time [210]. Rushworth et al. showed that mebendazole and docetaxel act synergistically to inhibit both in vitro and in vivo tumor growth of murine-derived PCC. This synergism is proposed to be through the inhibition of microtubule assembly at distinct areas, increasing the mitotic blockade of G2/M and thus, promoting cell death [150]. Niclosamide, another anthelminthic drug, has been shown to potently decrease expression of the androgen receptor variant AR-V7, which drives castration resistant PCa (CRPC). Consequently, it has potential to sensitize treatment to enzalutamide, an anti-androgen of the second generation, which is approved for use in treatment of patients with CRPC that are no longer responsive to docetaxel [151].

Niclosamide, an FDA-approved antihelminthic drug, exerts an anticancer effect in multiple types of cancer [211,212]. For example, niclosamide inhibits colorectal cancer cells’ liver metastases by downregulating S100A4 and blocking the Wnt/β-catenin signaling pathway [213,214]. It decreases lung cancer invasion by inhibiting the S100A4/NF-B/MMP9 axis: 350 reduces both the breast cancer cell pulmonary metastases and metastatic potential of lapatinib-resistant breast cancer cells by inhibiting the STAT3-FAK-Src axis [215] and epithelial-mesenchymal transition (EMT) [216], respectively. Furthermore, it inhibits the IL6-STAT3-AR signaling pathways in enzalutamide-resistant advanced PCa cells, reversing drug resistance and cellular invasion [217].

#### 3.2.6. Anti-Retroviral Drugs

Nelfinavir, a protease inhibitor used in HIV combination therapy, has shown potential to be repurposed in cancer therapy [152]. Guan et al. demonstrated that nelfinavir and its analogues help combat CRPC by directly inhibiting Site-2 Protease (S2P) cleavage and Regulated Intramembrane Processing (RIP) [153].

#### 3.2.7. Anti-Microbial Drugs

Minocycline, a semi-synthetic tetracycline derivative, was initially given FDA-approval for acne and some STDs, but it was later shown to have other non-antibiotic beneficial properties, including usage in inflammatory diseases [218]. Lokeshwar showed that chemically modified tetracyclines, particularly CMT-3, was able to promote apoptosis in PCa cell lines via activation of caspase-3 and caspase-9 [154].

Antifungal itraconazole, which has a high safety profile, has been noted for its anti-angiogenesis properties [219,220]. It inhibits mTOR, which reduces angiogenesis through the cholesterol trafficking route [221]. Itraconazole binds to the sterol-sensing domain of NPC1 and targets the mitochondrial protein VDAC1 to regulate AMPK and MTOR, resulting in the suppression of cell proliferation and angiogenesis [222]. In addition, it downregulates the PDGF/PI3K/Akt/mTOR pathway in infantile hemangioma [223]. A non-comparative, randomized, phase II study was conducted to evaluate the antitumor efficacy of two doses of oral itraconazole in men with metastatic PCa. Results showed that high-dose itraconazole (600 mg/day) has a modest antitumor activity in men with metastatic CRPC [219].

#### 3.2.8. Anti-Malarial Drugs

Artemisinin (ARS) is a well-known antimalarial medication that is used to treat about 600 million cases of malaria every year [224]. According to recent research, ARS and its derivatives exhibit anticancer activity as they promote non-apoptotic programmed cell death [225,226,227]. For example, artesunate (ART)-based drugs increased ROS production and favored lysosomal over autophagic ferritin degradation in cancer cells [228]. 

Similarly, chloroquine (CQ) and its derivative, hydroxychloroquine (HCQ), are antimalarial drugs that also treat several diseases, including rheumatoid arthritis, discoid lupus erythematosus, and systemic lupus erythematosus [229,230]. CQ or HCQ, the only FDA-approved autophagy flux inhibitors, have been used to treat pancreatic and other cancers [231,232]. 

Quinacrine, an antimalarial drug discovered in the 1920s, aids in the treatment of a variety of malignancies, owing to its ability to activate the p53 gene [233]. Mainly, quinacrine induces p53 expression by facilitating the chromatin transcription (FACT) protein complex, which is trapped onto the chromatin, thereby inhibiting CK2-mediated phosphorylation of p53 [234,235].

#### 3.2.9. Others

Zoledronic acid (ZA), a member of the bisphosphonates class of drugs, inhibits bone resorption via inhibition of osteoclast activity, hence its role in treatment of osteoporosis [236]. This repurposed drug has been approved for clinical use in treating PCa. Corey et al. demonstrated the anti-PCa effects of ZA, both in vivo by decreasing metastatic potential and in vitro by inducing G1 arrest, proliferation decline, and apoptosis [155]. In regard to PCa, ZA inhibits osteoblastic and osteolytic metastasis [155], while it clinically reduces skeletal complications in PCa patients with bone metastases [237]. Although the treatment with celecoxib, a COX-2 inhibitor, in combination with ZA therapy, is considered complementary for the treatment of advanced or metastatic PCa, it did not improve the survival rate [238]. In contrast, the combination of ZA and docetaxel enhanced PCa patients’ survival [210]. Based on early clinical trials, ZA is typically given every three weeks; however, current evidence suggests that giving ZA every 12 weeks results in similar outcomes in men with CRPC bone metastases [239].

Valproic acid (VPA) is used in the treatment of epileptic, bipolar, and schizophrenic disorders by inhibiting class I histone deacetylases (HDAC) [240]. Xia et al. demonstrated that chronic VPA administration results in reduced PCa cellular proliferation and upregulated caspase-2 and caspase-3 activity [156].

Mifepristone, an anti-progesterone with potent progesterone receptor affinity, is used for pregnancy termination. Etreby et al. showed mifepristone’s anti-cancer effects in both androgen sensitive and insensitive PCa, via induction of TFG-β-1, a pro-apoptotic transcription factor [157]. In fact, mifepristone’s anticancer activity is known for its ability to inhibit tumor growth via blocking the overexpressed cell surface receptors, such as progesterone, estrogen, and glucocorticoid receptors, in CRPC cells [241]. With combined estrogen treatment, anti-progesterone delivery to PCa cells suppressed the development of androgen-insensitive PCa in vivo [242]. The anti-tumor activity of mifepristone was reported against multiple cancer types, including androgen-sensitive and androgen-insensitive PCa [243]. In addition, a phase II study, performed by Taplin et al., suggested that the combination therapy of mifepristone with corticosteroids, ketoconazole, or 5-α reductase inhibitors might prevent the compensatory surge in adrenal androgens in patients with CRPC [244].

Disulfiram was first employed in the rubber vulcanization process, but it has been utilized as an alcohol-aversion medication to treat alcoholism [245,246]. Disulfiram’s mechanisms of action are closely linked to the acetaldehyde dehydrogenase (ALDH)-related cellular metabolic processes. For example, disulfiram-mediated acetaldehyde metabolism is a viable therapeutic target for BRCA1/2-deficient cancer cells [247]. ALDH positive atypical teratoid/rhabdoid tumor cells also had their metabolism decreased by disulfiram. Furthermore, because of its ability to block ALDH, disulfiram inhibits formaldehyde oxidation in cancer cells, resulting in cell death [248,249]. In addition, ALDH maintains the stemness of cancer cells, which explains disulfiram’s anticancer action in PCSCs [250,251]. Disulfiram serves as a DNA methyltransferase (DNMT1) inhibitor that restores tumor suppressor genes and DNA demethylation in PCa cells. Mainly, it reduces 5-methyl cytosine (5meC) content and methylation in APC and RARB gene promoters [252]. Hence, in a pilot trial for recurrent PCa, Schweizer and colleagues tested for disulfiram in epigenetic therapy via evaluating 5meC content in peripheral blood mononuclear cells (PBMC) [253]. Additionally, enhanced reduction in tumor growth was obtained upon the combination of disulfiram with Cu^2+^ in CRPC xenografts [254]. In addition, diocarb (disulfiram metabolite) and copper complex may potentially target NPL4 protein to regulate protein turnover of tumorigenesis, promoting stress-response pathways [255].

Rapamycin has been repurposed for cancer therapy after being authorized as an immunosuppressant for kidney transplantation [256] and an anti-restenosis drug [257,258]. Rapamycin inhibits mTORC1 by binding to the FRB domain of mTOR and hence its use in cancer treatment [259]. Treatment with rapamycin reduced leukemic progenitor cells in patients with acute myeloid leukemia [260,261]. It also demonstrated efficient antitumor activity in patients with drug-resistant chronic myelogenous leukemia, with very minor adverse effects in the majority of cases [262,263]. A study by our group demonstrated that Rapamycin is effective in the in vitro treatment of glioblastoma and neuroblastoma by targeting their CSC population [264]. 

Thalidomide, a glutamic acid derivative, was used as a sedative in 1957 and to treat morning sickness in pregnant women [265]. Nowadays, thalidomide is widely recognized as an antiangiogenic agent that inhibits VEGF, bFGF, tumor necrosis factor-alpha (TNF-α), and various other pro-angiogenic factors [266]. 

Interestingly, a study by our group assessed the in vitro and in vivo antitumor properties and mechanisms of action of ST1926 synthetic retinoids in targeting the cancer stem-like cells population of human PCa. Our results revealed that ST1926 substantially reduced proliferation of PCa cells and induced cell cycle arrest, p53-independent apoptosis, and early DNA damage. It also significantly reduced prostate spheres’ formation ability in vitro, denoting sufficient eradication of the self-renewal ability of the highly androgen-resistant CSCs [267].

## 4. Clinical Trials of Drugs Repurposed in PCa

Typically used cancer therapies are known to carry severe side effects. Findings of medications that are not typically used for this disease is encouraging for several reasons: they are already discovered, most have been on the market for a long time, proving their safety, they can help with diagnosing or treating, many are cost-effective when compared to chemotherapy or radiation, and they may improve quality of life, with minimal side effects. This applies to PCa.

We have shown that many drugs have been tested in preclinical studies to assess their anti-tumor activity in PCa. However, many of those drugs have already made it into clinical trials. Categories of those medications include anti-inflammatory (both steroidal and non-steroidal), anti-arrhythmic, anti-diabetic, anti-helminthic, anti-fungal, antibiotics, lipid-lowering agents, anticoagulants, bisphosphonates, anti-parasitics, immunomodulators, growth factor inhibitors, farnesyltransferase inhibitors, ribonucleotide reductase inhibitors, polysaccharide, retinoid derivatives, hormones, sugars, cyclic peptides, alpha-keto acids, fatty acids, vitamins, and chemical elements (Table 2).

## 5. Conclusions and Future Directions

Prostate cancer cells exploit various cell signaling channels to become castration resistant. Blockade of these channels can terminate or slow the growth of resistant tumors. A plethora of drugs designed for other diseases target the channels used by CRPC. Thus, repurposing these drugs to treat PCa will increase the treatment repertoire. Repurposing offers the advantage of bypassing Phase I and II clinical trials and, thus, speeds up drug approval. Many clinical trials have been designed that repurpose drugs to treat PCa, and more will follow. As electronic health records become more integrated, and artificial intelligence and precision medicine advance, more drugs will be strategically repurposed to treat PCa. Drugs that target the nuances of patient-specific tumors will be identified and hopefully improve patient life expectancy, cost of treatment, and quality of life. Functional genomics is also widely used to map cancer dependencies and identify therapeutic targets in cancer. This includes genetic screens based on CRISPR/Cas9 technology [69,70,71], reverse genetic screens, and chemogenomic screens [72]. In the era of personalized medicine, drug repurposing presents an opportunity and should be implicated, using computational genomic and proteomic technologies, to guide clinicians in their decision-making regarding patient treatment. Studies should continue reviewing the current trends of repurposing, as this is an efficient and valuable practice for drug discovery.

## Figures and Tables

**Table 2:** Summary table of the drugs that have been repurposed to be used in prostate cancer in clinical trials.

Drug Category	Clinical Trial Title	Number ClinicalTrials.gov ID	Phase	Study Start Date	Estimated Enrollment	Status	Intervention
Anti-diabetic	Metformin Hydrochloride as First-Line Therapy in Treating Patients with Locally Advanced or Metastatic PCa	NCT01243385	II	1 December 2010	44	Completed	Metformin
Anti-diabetic	Castration Compared to Castration Plus Metformin as First Line Treatment for Patients with Advanced PCa	NCT01620593	II	1 April 2011	41	Completed	Metformin
Anti-diabetic	Prevention of Metabolic Syndrome and Increased Weight Using Metformin Concurrent to ADT and Radiotherapy for Locally Advanced PCa	NCT01996696	II	1 September 2014	104	Recruiting	Metformin
Anti-diabetic	Repurposing Metformin as Anticancer Drug in Advanced PCa (Mansmed)	NCT03137186	II	1 January 2017	120	Unknown	Metformin
Anti-diabetic	Study of Metformin Plus Oligomeric Procyanidin Complex for Pharmacologic Manipulation of AGE Levels in PCa Patients	NCT03465345	Ib	1 July 2018	0	Withdrawn	Metformin + oligomeric procyanidin complex
Anti-diabetic	Drug-Drug Interaction of SHR3680 with Digoxin, Rosuvastatin Calcium and Metformin Hydrochloride	NCT04621669	I	1 November 2020	18	Not yet recruiting	Metformin hydrochloride + SHR3680 + digoxin + rosuvastatin
Anti-hyperlipidemic	Impact of Adjuvant Statin Therapy in Patients who Underwent Radical Prostatectomy for Locally Advanced PCa	NCT01759836	II	1 October 2012	354	Unknown	Atorvastatin vs. placebo
Anti-parasitic	Low, Intermediate, or High Dose Suramin in Treating Patients with Hormone-Refractory PCa	NCT00002723	III	1 January 1996	390	Completed	Suramin
Anti-parasitic	Combination Chemotherapy with Suramin Plus Doxorubicin in Treating Patients with Advanced Solid Tumors	NCT00003038	I	1 October 1997	20	Completed	Doxorubicin + suramin
Anti-parasitic	Akt Inhibitor MK2206 and Hydroxychloroquine in Treating Patients with Advanced Solid Tumors, Melanoma, Prostate or Kidney Cancer	NCT01480154	I	1 November 2011	62	Active, not recruiting	Hydroxycloroquine + Akt inhibitor MK2206
Anti-helminthic	Enzalutamide and Niclosamide in Treating Patients with Recurrent or Metastatic CRPC	NCT03123978	I	1 January 2017	12	Recruiting	Enzalutamide + niclosamide
Anti-helminthic	Niclosamide and Enzalutamide in Treating Patients With CRPC	NCT02532114	I	1 December 2015	5	Completed	Niclosamide + enzalutamide
Anti-fungal	Hormonal Therapy and Chemotherapy Followed by Prostatectomy in Patients with PCa	NCT02494713	II	1 October 2015	4	Terminated	Degarelix + Doxorubicin + Ketoconazole + Docetaxel + Estramusine
Anti-fungal	Hormone Therapy Plus Chemotherapy as Initial Treatment for Local Failures or Advanced PCa	NCT02560051	II	1 November 2015	19	Terminated	Doxorubicin + ketoconazole + docetaxel + estramustine
Antiviral	A Study of Aplidin (Plitidepsin) in Subjects with Advanced PCa	NCT00780975	II	1 February 2005	8	Terminated	Plitidepsin
NSAID	Dexamethasone, Aspirin, and Diethylstilbestrol in Treating Patients with Locally Advanced or Metastatic PCa	NCT00316927	III	1 December 2002	260	Completed	Dexamethasone + aspirin vs. dexamethasone + diethylstilbestrol + aspirin
Steroid	Combination of Docetaxel + Estramustine + Hydrocortisone Versus Docetaxel + Prednisone in Patients with Advanced PCa (PROSTATA)	NCT00705822	III	1 August 2006	54	Terminated	Docetaxel + prednisone vs. docetaxel + estramusine + hydrocortisone
Steroid	Docetaxel, Prednisone, and Vatalanib in Treating Patients with Advanced PCa	NCT00293371	I/II	1 September 2006	6	Terminated	Docetaxel + prednisone + vatalanib
Steroid	A Safety and Efficacy Study of Abiraterone Acetate in Participants with Advanced PCa who Failed ADT and Docetaxel-Based Chemotherapy	NCT00474383	II	1 November 2006	47	Completed	Abiraterone acetate + glucocorticoid
Steroid	An Open-Label Study of YM155 + Docetaxel in Subjects with Advanced Hormone Refractory PCa and Other Solid Tumors	NCT00514267	I/II	1 May 2007	32	Completed	YM 155 + docetaxel + prednisone vs. YM 155 + docetaxel
Steroid	An Efficacy and Safety Study of Abiraterone Acetate and Prednisone in Participants with PCa Who Failed ADT and Docetaxel-Based Chemotherapy	NCT00485303	II	1 June 2007	58	Completed	Abiraterone acetate + prednisone
Steroid	AMG 386 and Abiraterone for Advanced PCa	NCT01553188	II	1 February 2012	36	Completed	Abiraterone + prednisone vs. abiraterone + prednisone + AMG
Steroid	Cabozantinib Plus Docetaxel and Prednisone for Advanced PCa	NCT01683994	I	1 September 2012	49	Completed	Carbozantinib + docetaxel + prednisone
Steroid	Reducing Dexamethasone Around Docetaxel Infusion (REDEX)	NCT02776436	I	1 January 2016	46	Active, not recruiting	Dexamethasone
Steroid	Cognitive Effects of AR Directed Therapies for Advanced PCa	NCT03016741	IV	1 March 2017	100	Recruiting	GnRH agonist/antagonist + prednisone + abiraterone acetate vs. GnRH agonist/antagonist + enzalutamide
Steroid	Intermittent ADT for Stage IV Castration Sensitive PCa	NCT03511196	1b	1 September 2018	17	Active, not recruiting	ADT + abiraterone + prednisone
Steroid	A Study of Nivolumab or Placebo in Combination with Docetaxel in Men with Advanced CRPC (CheckMate 7DX)	NCT04100018	III	1 February 2020	984	Recruiting	Nivolumab + prednisone + docetaxel vs. placebo
Immunosuppressive drug	Sirolimus Before Surgery in Treating Patients with Advanced Localized PCa	NCT00311623	I/II	1 August 2006	32	Completed	No intervention vs. low dose rapamycin vs high dose rapamycin
Synthetic vitamin D analogue	Paricalcitol in Treating Patients with Advanced PCa and Bone Metastases	NCT00634582	II	1 January 2009	2	Terminated	Paricalcitol
Vitamin D analogue	Effect of CTAP101 Capsules on Ca/iPTH in Advanced Breast/PCa Patients Treated with Denosumab/Zoledronic Acid	NCT02274623	I	1 December 2014	33	Completed	CTAP101 capsules (vitamin D analogue)
Vitamin D	Changes in Bone Mineral Density and Fracture Risk in Patients Receiving ADT for PCa	NCT00536653	N/A *	1 October 1999	618	Completed	Bicalutamide + calcium/vitamin D supplementation vs. LHRH agonists + calcium/vitamin D supplementation
Bisphosphonate	Study of Zoledronic Acid for Patients with Hormone-sensitive Bone Metastases from PCa	NCT00242567	III	1 December 2005	522	Completed	Zoledronic acid
Bisphosphonate	Zoledronate in Preventing Osteoporosis and Bone Fractures in Patients with Locally Advanced Nonmetastatic PCa Undergoing Radiation Therapy and Hormone Therapy	NCT00329797	III	1 March 2006	109	Completed	Calcium + zoledronic acid + radiation therapy + LHRH + vitamin D vs. calcium + radiation therapy + LHRH + vitamin D
Bisphosphonate	Study of Denosumab vs. Zoledronic Acid to Treat Bone Metastases in Subjects with Advanced Cancer or Multiple Myeloma.	NCT00330759	III	1 June 2006	1779	Completed	Denosumab + zoledronic acid
Somatostatin analogue	Effects of Octreotide Acetate on Circulating Levels of Chromogranin A in Advanced PCa Patients	NCT00166725	II	1 February 2004	40	Completed	Octreotide acetate
Anticoagulant	Standard Therapy with or Without Dalteparin in Treating Patients with Advanced Breast, Lung, Colorectal, or PCa	NCT00003674	III	1 December 1998	141	Completed	Dalteparin
Anticoagulant	Effects of Nadroparin in Patients with Lung, Pancreas or PCa	NCT00312013	III	1 May 2006	503	Completed	Nadroparin
Synthetic retinoid	Fenretinide In Treating Patients with Advanced or Metastatic Hormone-Refractory Prostate Cancer	NCT00077402	II	1 February 2004	50	Completed	Fenretinide
Other	Preoperative Thalidomide Followed by Radical Retropubic Prostatectomy in Select Patients with Locally Advanced PCa	NCT00038181	II	5 October 2000	18	Completed	Thalidomide preoperatively
Other	Trial of Docetaxel-Samarium in Patients with Hormone-Refractory Advanced PCa	NCT00126230	II	1 January 2004	55	Terminated	Docetaxel-samarium
Other	Samarium-153 With Neoadjuvant Hormonal and Radiation Therapy for Locally Advanced PCa	NCT00328614	I	1 March 2003	32	Completed	Samarium + hormonal and radiation therapy
Other	Study of Chitosan for Pharmacologic Manipulation of AGE Levels in PCa Patients	NCT03712371	Ib/2	1 January 2019	45	Recruiting	Chitosan
Other	Study of Pharmacologic Manipulation of AGE Levels in PCa Patients Receiving ADT	NCT02946996	II	1 December 2016	45	Recruiting	AGE
Other	A Phase I/II Trial of 2-Deoxyglucose (2DG) for the Treatment of Advanced and Hormone Refractory PCa	NCT00633087	I/II	1 March 2011	12	Terminated	2-deoxyglucose
Other	Omega-3 Fatty Acids in Treating Patients with Advanced PCa	NCT00996749		1 May 2011	0	Withdrawn	Omega-3 PUFA supplementation
Other	Hyperpolarized C-13 Pyruvate as a Biomarker in Patients with Advanced Solid Tumor Malignancies	NCT02913131	I/II	1 October 2016	20	Terminated	Pyruvate
Other	Nanoparticle Albumin-Bound Rapamycin in Treating Patients with Advanced Cancer with mTOR Mutations	NCT02646319	I	1 January 2016	2	completed	Rapamycin
Other	Enzalutamide/Leuprolide ± Abiraterone/Pred in PCa	NCT02268175	II	1 October 2014	75	Active, not recruiting	Enzalutamide + leuprolife + abiraterone acetate + prednisone vs. enzalutamide + leuprolide
Other	Study to Compare the Effects of Repeated Doses of an Investigational New Drug and a Placebo on Appetite in Advanced PCa and Anorexia	NCT04803305	I	1 May 2021	40	Recruiting	PF-06946860
Other	Study of XL102 as Single-Agent and Combination Therapy in Subjects with Solid Tumors	NCT04726332	I	1 February 2021	298	Recruiting	XL102 + fulvestrant + abiraterone + prednisone

Abbreviations: ADT: Androgen Deprivation Therapy; AGE: Advanced Glycation End products; AR: Androgen Receptor; CRPC: castration resistant prostate cancer; PCa: prostate cancer. * N/A: Not Applicable is used to describe trials without FDA-defined phases, including trials of devices or behavioral interventions.

## Data Availability

Not applicable.
